# The Safety and Acceptance of the PrePex Device for Non-Surgical Adult Male Circumcision in Rakai, Uganda. A Non-Randomized Observational Study

**DOI:** 10.1371/journal.pone.0100008

**Published:** 2014-08-21

**Authors:** Godfrey Kigozi, Richard Musoke, Stephen Watya, Nehemia Kighoma, James Nkale, Mary Nakafeero, Dan Namuguzi, David Serwada, Fred Nalugoda, Nelson Sewankambo, Maria Joan Wawer, Ronald Henry Gray

**Affiliations:** 1 Rakai Health Sciences Program, Entebbe, Uganda; 2 Department of Urology, Mulago Hospital, Kampala, Uganda; 3 School of Public Health, Makerere University College of Health Sciences, Kampala, Uganda; 4 Department of Epidemiology, Johns Hopkins University, Bloomberg School of Public Health, Baltimore, Maryland, United States of America; 5 College of Health Sciences, Makerere University, Kampala, Uganda; University of the Stellenbosch, South Africa

## Abstract

**Objectives:**

To assess the safety and acceptance of the PrePex device for medical male circumcision (MMC) in rural Uganda.

**Methods:**

In an observational study, HIV-uninfected, uncircumcised men aged 18 and older who requested elective MMC were informed about the PrePex and dorsal slit methods and offered a free choice of their preferred procedure. 100 men received PrePex to assess preliminary safety (aim 1). An additional 329 men, 250 chose PrePex and 79 chose Dorsal slit, were enrolled following approval by the Safety Monitoring Committee (aim 2). Men were followed up at 7 days to assess adverse events (AEs) and to remove the PrePex device. Wound healing was assessed at 4 weeks, with subsequent weekly follow up until completed healing.

**Results:**

The PrePex device was contraindicated in 5.7% of men due to a tight prepuce or phimosis/adhesions. Among 429 enrolled men 350 (82.0%) got the PrePex device and 79 (18.0%) the dorsal slit procedure. 250 of 329 men (76.0%) who were invited to choose between the 2 procedures chose Prepex. There were 9 AEs (2.6%) with the PrePex, of which 5 (1.4%) were severe complications, 4 due to patient self-removal of the device leading to edema and urinary obstruction requiring emergency surgical circumcision, and one due to wound dehiscence following device removal. 71.8% of men reported an unpleasant odor prior to PrePex removal. Cumulative rates of completed wound healing with the PrePex were 56.7% at week 4, 84.8% week 5, 97.6% week 6 and 98.6% week 7, compared to 98.7% at week 4 with dorsal slit (p<0.0001).

**Conclusion:**

The PrePex device was well accepted, but healing was slower than with dorsal slit surgery. Severe complications, primarily following PrePex self-removal, required rapid access to emergency surgical facilities. The need to return for removal and delayed healing may increase Program cost and client burden.

## Introduction

Medical male circumcision (MMC) reduces male HIV acquisition by 50–60% [1]–[3] and has been recommended by UNAIDS/WHO as a component of HIV prevention [4]. However, progress with scale up of MMC services in 14 priority countries in sub-Saharan Africa has been suboptimal [5], and there is a need to improve access to and the efficiency of MMC services. One obstacle to MMC scale up in adolescents and adults is the time and provider skills required for conventional surgical procedures (forceps guided, dorsal slit and sleeve circumcision) all of which entail a surgical time of 15–45 minutes (depending on provider experience), aseptic conditions and skill in hemostasis and suturing for wound closure. Two devices, the Shang Ring [6] and the PrePex device [7]–[10] offer potential advantages in reduction of procedure time which could increase throughput of clients, a lower requirement for asepsis and the procedures can be performed by less skilled providers [7], [8], [10] which could facilitate task shifting and sharing. Both devices must be removed 5–9 days after placement.

The Shang Ring requires injectable local anesthesia and primary surgical removal of the foreskin, and the procedure takes ∼5 minutes [6]. We previously reported findings on the safety and acceptability of this MMC method [11] The PrePex device is applied under topical anesthesia because the foreskin remains intact and is compressed by radial elastic pressure leading to distal necrosis thus shortening procedure time. The method requires secondary removal of necrotic skin at 5–9 days post-placement. Published studies of the PrePex device in Rwanda suggest that the time required for placement is ∼3 minutes [7]–[9], that lower cadre personnel such as nurses can safely place the device [7], and that adverse events are infrequent. The World Health Organization Technical Advisory Group (WHO TAG) reviewed eight published and unpublished studies of the PrePex device in January, 2013, and concluded that in ∼7% of clients the PrePex device could not be used because a tight foreskin precluded placement, that adverse events were ∼1.7% and that severe adverse events, mainly due to displacement or self-removal of the elastic ring, required urgent surgical intervention in 0.4% of cases [12]. The WHO TAG provided prequalification for use of the PrePex in men aged 18 or older, but recommended that skilled surgical back up was available to manage severe complications [12]

We conducted a safety and acceptability study of the PrePex device in rural Rakai District of southwestern Uganda.

## Methods

### Ethics Statement

The study was reviewed and approved by Institutional Review Boards in Uganda (the Scientific and Ethics Committee of the Uganda Virus Research Institute and the Uganda National Council for Science and Technology), and the U.S.A. (the Johns Hopkins University, Bloomberg School of Public Health Institutional Review Board). Permission to import the PrePex device for research was provided by the Uganda National Drug Authority, and the study was monitored by a Safety Monitoring Committee (SMC.) Under the study protocol, enrollment was paused after the first 50 and 100 PrePex device placements and findings were reviewed by the SMC prior to continuation of enrollment.

This implementation research study was designed and conducted as an observational study between November 2012 and June 2013 in Rakai district, Uganda. Participant enrollment was done between November 2012 and May 2013. The last follow-up visit was conducted in June 2013. Men, who requested free MMC services from the Rakai Health Sciences Program (RHSP), were informed about the dorsal slit and PrePex methods of circumcision, and offered free HIV Counseling and Testing (HCT). Uncircumcised HIV-negative men aged 18 years and older were invited to enroll in the study and asked to provide written informed consent. One hundred (100) men were enrolled and PrePex placed to assess preliminary safety of the PrePex device (aim 1). The safety monitoring committee approved continuation of enrolment (aim 2) to enable assessment of safety and acceptability of PrePex. The consent form explained the nature of the two MMC procedures, known risks and benefits, and their ability to select their procedure of choice. Consenting men were provided with further detailed information and asked to nominate their preferred method of MMC and to give reasons for their preference. All men were screened for contraindications to their method of choice. Men with symptoms or signs of genitourinary infection (discharge, ulceration or dysuria) were treated and MMC was deferred until the problem was resolved. Men with anatomic abnormalities were referred for assessment by the consultant urologists (SW and DN).

MMC was performed by trained clinical officers (equivalent to a physician's assistant) and registered nurses. The dorsal slit was performed as described in the WHO manual for male circumcision under local anesthesia ^13^ The PrePex device was placed according to the manufacturer's instructions (Circ MedTech Ltd, Israel.) The diameter of the penis at the coronal sulcus was determined using the manufacturer's sizing device and an appropriately sized device was selected. Lignocaine 5% gel prepared by a Rwanda team of instructors, was applied to the inner surface of the foreskin and the firm inner ring inserted at the level of the coronal sulcus. The elastic O-ring was then applied to the external surface of the foreskin using a placement device, to compress the foreskin hence blocking blood flow to the distal preputial tissue. Participants were observed for a minimum of one hour post-placement and pain was assessed using a visual analogue scale (VAS). Men were provided with acetaminophen to alleviate any post-placement pain. Men were then followed up seven days post-placement and the ring and necrotic foreskin were removed per manufacturer's instructions. Pain control during removal used a 10% Cetacaine spray which was applied between the residual necrotic prepuce and the coronal sulcus. Pain during or after removal was assessed by the VAS. Active follow up was used to trace men who failed to come for the scheduled device removal visit.

Participants were then followed up at 4 weeks post-device placement to assess wound healing. Complete wound healing was defined as an intact scar with no scab formation. Men with incomplete wound healing were followed up at weekly intervals until complete wound healing was certified. Unscheduled visits could occur at any time in case of adverse events or other concerns, and participants could contact the Program using a cell phone hot line.

Interviews were conducted at enrollment to ascertain sociodemographic characteristics and sexual behaviors. At each follow up visit, interviews were conducted using standard questionnaires to determine symptoms suggestive of adverse events (AEs), resumption of intercourse, condom use and sexual satisfaction/dysfunction among those who had resumed intercourse. Pain was assessed using a VAS, and pain scores of 7 or above lasting more than 2 minutes were classified as adverse events. PrePex recipients were also asked about post-placement odor from the necrotic foreskin and whether this interfered with their normal activities of daily living.

Adverse events (AEs) were predefined based on classification provided by the manufacturer and the Rwandan Team. Severity was graded as mild if no treatment was needed, moderate if non-surgical treatment was indicated, and severe if surgical intervention or hospitalization was required. Definitions of AEs can be provided on request.

### Statistical methods

We present results from the PrePex training phase in which 136 devices were provided by trainees and during the research phase of the study when 350 PrePex devices and 79 dorsal slit MMCs were provided. For the latter phase, we tabulated the number of men who declined to enroll and reasons for non-enrollment, including contraindications to use of the device. The sociodemographic and behavioral characteristics at enrollment were compared between those choosing the PrePex or dorsal slit methods using chi-square tests. Follow up at scheduled visits, severity of pain at placement and removal, experience of unpleasant odor with the PrePex, occurrence of adverse events and the proportions with completed wound healing at 4–7 weeks post-MMC were assessed. A two sample test of proportions was used to compare the proportion with completed wound healing between those who received the PrePex versus those circumcised using the dorsal slit method.

## Results

We present the results separately for the training phase and for the main study.

### Training phase

Training in PrePex placement and removal was conducted and certified by PrePex Product Specialists from Rwanda (Dr.Ngeruka M. Leon, Dr.Sibomana Alphouse, Amuhinde Jacpus and Lior Levert) following the manufacturer's guidelines. Seven personnel were trained, and each conducted an average of 13 PrePex circumcisions. There were 136 device placements during training, and 135 (99.3%) of men were followed up at 7 days for device removal. Post-placement pain was minor, but some discomfort (e.g. some mild pain on movement) while wearing the device during the first week was reported by 26.7% of men. There were 9 mild adverse events (6.7%), eight (5.9%) were due to pain with a VAS score ≥7 at time of device removal, and one was due to self-removal of the device because of parental instruction. The removal of the device was without complications and dorsal slit surgery was provided as a service. An unpleasant odor was reported by 77.8% of men, and 30.5% said this interfered with their daily activities.

Follow up at 4 weeks was completed by 122 men (89.7%), and complete wound healing was certified in 49.2%. Cumulative wound healing was 90.2% at week 5, 98.9% at week 6, and 100% by the 7^th^ week.

### Study Phase

**Figure 1:**
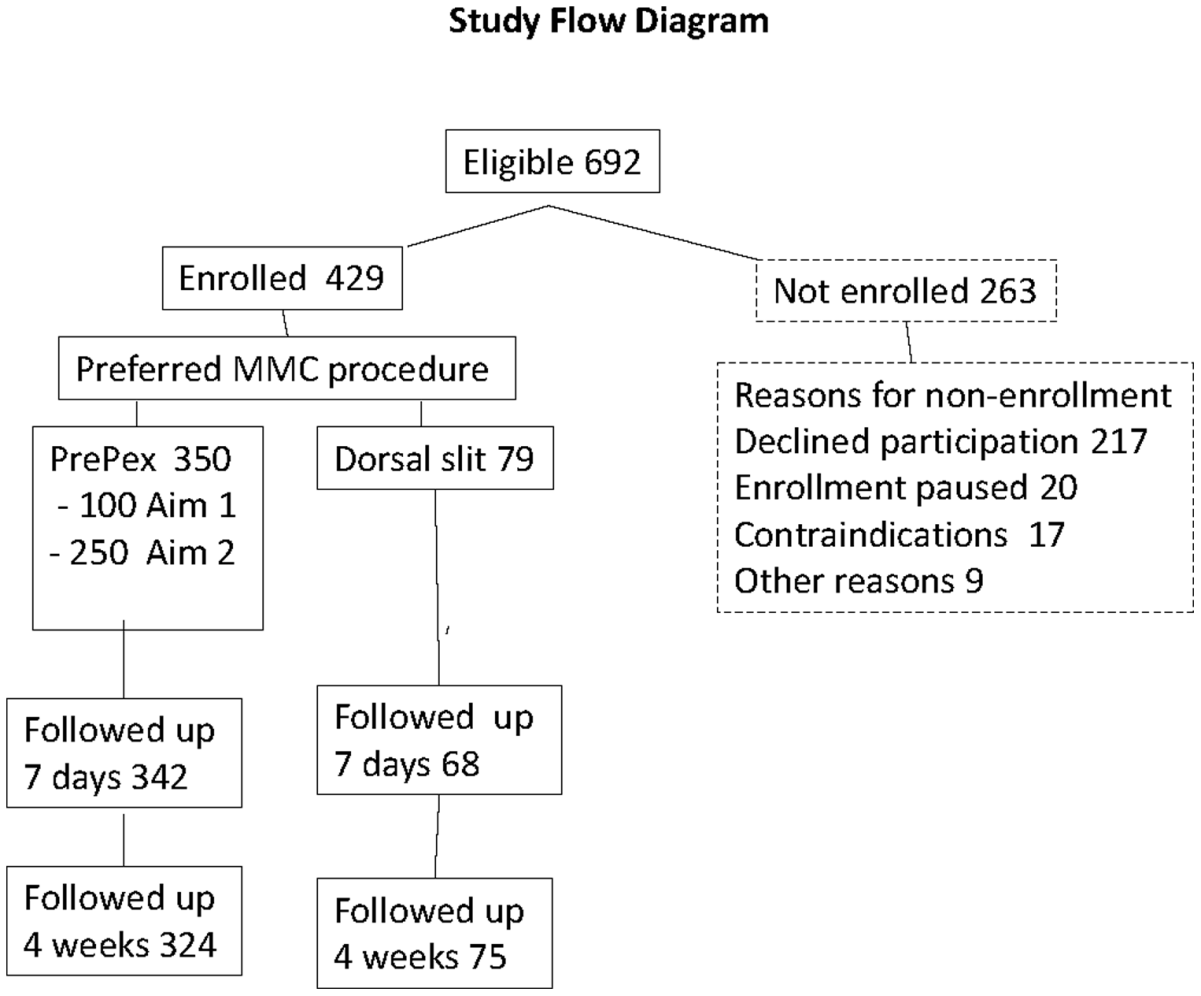
Figure 1 shows the study flow chart.

One hundred (100) men were enrolled in the preliminary safety stage (aim 1) and all got PrePex. There were 592 potentially eligible men for aim 2 of whom 329 (55.6%) were enrolled into the study; 250 (76.0%) chose the PrePex device and 79 (24.0%) selected the dorsal slit procedure (Figure 1). Among the 263 men who did not enroll, the predominant reasons for non-enrollment were inability to adhere to the follow up schedule (56.7%), lack of interest in the study (25.9%) and protocol determined pauses of enrollment (7.6%). Contraindications to placement of the PrePex device were found in 17 participants (6.5%) and included narrowing of preputial opening (5.7%) due to severe phimosis, tight foreskin and adhesions.

The characteristics and behaviors at enrollment were similar among those choosing the PrePex or dorsal slit (Table 1), and the majority of participants were young men aged between 18–24 years. Among PrePex recipients, the predominant device sizes were B (28.6%), C (34.8%) and D (18.0%). Pain after Prepex placement was mild and was reported by 2.9% of men at 30 minutes, 4.9% at one hour and 2.3% at two hours post-device placement.

**Table 1:** Characteristics and behaviors of men who received the PrePex or dorsal slit circumcision.

Characteristics of men	PrePex(N = 350)		Dorsal slit(N = 79)		P value
	Number	Col %	Number	Col %	
***Total***	***350***	***100***	***79***	***100***	
**Age**					0.963
18–25	233	66.6	51	64.6	
25–29	51	14.6	13	16.5	
30–34	28	8	6	7.6	
35–39	10	2.9	3	3.8	
40+	28	8	6	7.6	
**Education**					0.496
Primary	216	61.7	52	65.8	
Secondary or above	134	38.3	27	34.2	
**Marital status**					0.262
Currently married	139	39.7	26	32.9	
Not married	211	60.3	53	67.1	
**Sex in the past year**					0.501
Yes	261	74.6	56	70.9	
No	89	25.4	23	29.1	
**Sex partners in the past year**					0.197
**Among the sexually active**					
One partner	140	40	37	46.8	
2 or more partners	121	34.6	19	24.1	
**Non-marital partners in the past year among the sexually active**					0.571
Unmarried	89	25.4	22	27.8	
Married 1 non-marital partner	82	23.4	23	29.1	
Married, 2+ non-marital partners	86	24.6	15	19	
**Condom use among the sexually active**					0.731
None	61	17.4	10	12.7	
Inconsistent	26	7.4	5	6.3	
Consistent	28	8	7	8.9	
**Alcohol use**					0.636
Yes	116	33.1	24	30.4	
No	234	66.9	55	69.6	

Follow up at 7 days was completed by 97.7% of PrePex recipients (342/350) and 86.1% of dorsal slit recipients (68/79). Among the 342 PrePex clients seen at day 7, 14.3% (49) reported some discomfort over the preceding week. Virtually all men reported some pain at time of device removal but none reported severe pain scored ≥7 on the VAS. An unpleasant smell during the first week after PrePex placement was reported by 71.8% of men, 8.6% said this odor affected activities of daily living and 10.2% said the odor was noticed by other people in the vicinity. Follow up for the scheduled 4 week visit was 93.1% (326/350) among PrePex and 94.9% (75/79) among dorsal slit recipients. Wound healing was certified as complete in 56.7% (185/326) PrePex and 98.7% (74/75) dorsal slit circumcisions (p<0.0001). The cumulative rates of completed wound healing with the PrePex device were 84.8% at week 5, 97.6% at week 6 and 98.6% at week 7.

**Figure 2:**
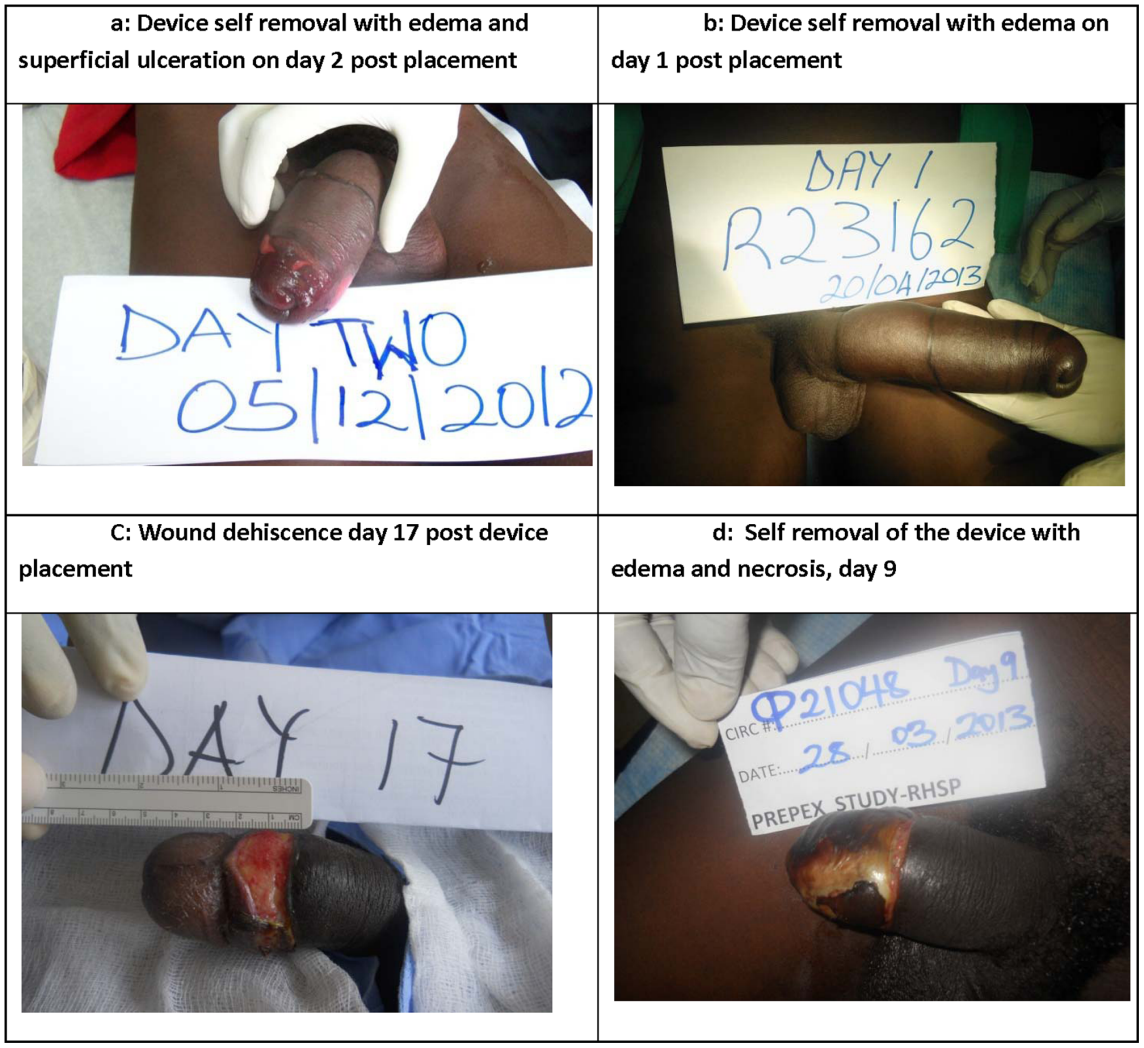
Figure 2 shows photographs of selected adverse events with the PrePex device. Figure 2a, device self removal with edema and superficial ulceration on day 2 post placement. Figure 2b, device self removal with edema on day 1 post placement. Figure 2C, wound dehiscence on day 17 post device placement. Figure 2d, self removal of the device with edema and necrosis on day 9.

Adverse events with the PrePex device are tabulated in Table 2. There were a total of 9 adverse events (2.6%), 4 were mild events (1.1%) and five were severe adverse events (1.4%) which are summarized in Table 3, and selected severe adverse events are shown in Figure 2 a–d with written consent of participants. Among the mild AEs, two participants removed the device without complications and did not request surgical circumcision as a service, one had device displacement and one had edema distal to the circumcision scar Four severe events were due to premature non-medical removal of the device and in 3 cases the participant removed the device because of edema, pain, or difficulty voiding. All 4 SAEs required sleeve circumcision and all subsequently resolved. One participant had the device removed on the 7^th^ day without complications, but subsequently experienced wound dehiscence requiring surgical repair. It is noteworthy that 7 participants (2.0%) removed the device themselves, and four experienced serious complications (1.1%). No AEs were observed among 79 dorsal slit recipients.

**Table 2:** Number of adverse events with PrePex circumcision.

Adverse events and severity	PrePex (N = 350)	
	Number	%
**Mild AEs**		
Device displacement	1	0.3
Device removed by participant, no complications	2	0.6
Edema	1	0.3
All mild AEs	4	1.1
**Moderate AEs**	0	na
**Severe AEs**		
Device removed by participant with complications	4	1.1
Wound dehiscence following medical removal of device	1	0.3
All severe AEs	5	1.4
Total AEs	9	2.6

**Table 3:** Details of adverse events with the PrePex device during the study.

Severe Adverse event	Description
Device removal due to reported assault	The participant claimed he was assaulted and the elastic ring was removed by force. He had severe edema of the foreskin, hemorrhage and necrosis requiring surgical circumcision (Figure 2 a). The wound healed by the 5^th^ week post-placement and the problem was resolved.
Device removed by participant following complications	The participant had edema of the foreskin, discomfort and difficulty voiding the day after device placement. He removed the elastic ring and developed more severe edema, difficulty voiding and loss of sensation requiring surgical circumcision. The wound was healed by the 4^th^ week post-placement and the problem was resolved (Figure 2 b)
Device removed by participant due to complications	The participant had edema and difficulty voiding the day after device placement. He removed the elastic ring. He had severe edema of the foreskin and loss of sensation requiring surgical circumcision. The wound healed and the problem was resolved.
Device displacement and removal with wound dehiscence	Participant had an erection on the 4^th^ post-placement day leading to displacement of the outer ring and loss of the device. He returned on the 9^th^ post-placement day with a purulent granulating circumferential wound 0.5 cms wide requiring debridement, suturing and antibiotics. He missed follow up visits, but the wound was healed when observed on the 8^th^ week post-placement (Figure 2 d).
Wound dehiscence	Participant had an erection on the 1^st^ day after medical removal of the device resulting in mild bleeding for which he was treated with an injection, wound dressing and instructed to have salty dressing by a local practitioner. Despite the treatment he developed gaping of the wound. 10 days following onset of event he was examined and a 2 cm wide circumferential dehiscence with nice granulation was seen. He had secondary wound closure under local anesthesia. He was fully healed at 5^th^ week post placement (Figure *2* c).
**Mild Adverse Events**	
Device displacement	The device was displaced distally on the 3^rd^ day post-placement. The participant tried to reposition the device but it and the necrotic foreskin detached spontaneously without complications. The wound was healed by the 4^th^ week post-placement.
Device removal without complications	The participant removed the device on the day of placement after being told it would impair erectile function and fertility. There were no complications. Participant did not request to be circumcised.
Device removal without complications	The participant removed the device 3 hours after placement due to pain without complications. He requested circumcision using the PrePex method. Prepex was not used since it had not been approved to be used for service circumcision.
Edema	The participant had edema distal to the circumcision wound that took 7 months to resolve.

The mean time for device placement was 4.5 minutes (range 2–18 minutes), and the mean time required for removal was 4.3 minutes (range2–14) minutes. The mean time required for dorsal slit was 14.9 minutes (range 8–35 minutes).

## Discussion

The PrePex device was well accepted among study participants in this rural Ugandan population, and 76.0% (250/329) selected the PrePex device rather than dorsal slit surgery when offered a free choice of MMC method. It should however be noted that 263 (44.4%) eligible men were not enrolled in the study for various reasons the commonest being reluctance or inability to adhere to the study follow-up schedule or lack of interest in the study (figure 1). These opted for circumcision using the dorsal slit method via the routine MMC service program. This suggests that if PrePex is introduced in this population only ∼42.2% (250/592) men would receive circumcision using Prepex.

The time required for placement and removal of the device was substantially shorter than dorsal slit surgery, but there is need for a mandatory day 7 visit for device removal. The time for complete wound healing was slower with the PrePex than conventional surgery, as has been found in other studies. The need for a mandatory day 7 visit for device removal and need for additional follow up due to a longer healing time burdens the clients and increases the costs associated with PrePex.

The observed rate of moderate/severe adverse events (1.4%) was similar to that reported in other PrePex studies [7], [9], [13], and comparable to rates with the Shang Ring device (1.0%) and dorsal slit surgery (0.8%) in this population [11] However, we observed 7 cases of participant self-removal of the PrePex device (2.0%), and 4 of these cases resulted in severe complications requiring immediate surgery. One Rwandan study observed two cases of self-removal in 518 PrePex procedures (0.4%) [7] but another Rwandan study of 144 PrePex recipients reported no cases [9] Unpleasant smell was reported by 71.8% of clients with 10.2% reporting that the odor was noticed by people in their vicinity. This calls for measures to prevent or minimize the offensive odor since it can affect both clients and providers.

We have conducted two studies of MMC devices in Rakai, Uganda which allow comparison between the Shang Ring and PrePex devices in a single setting. A randomized trial was not possible because the manufacture was reluctant to sell the PrePex device for a comparative trial of the PrePex and Shang Ring. Therefore, we can not directly asses the relative advantages and disadvantages of the two devices. The Shang Ring requires local anesthesia injection and greater attention to asepsis because the foreskin is surgically removed, whereas the PrePex requires only topical anesthesia and the need for asepsis is less because there is no open surgical wound. We observed no severe adverse events with 500 Shang Ring procedures [11] and the WHO TAG reported none among 1,983 procedures [12], whereas we found a rate of 1.4% severe adverse events which required emergency surgery among 350 PrePex procedures. Thus, the risk of severe adverse events with the PrePex, mainly attributable to self-removal of the device, constitutes a potential disadvantage. An analogy can be made to a randomized trial of neonatal circumcision in Botswana in which rare but severe adverse events due to displacement of the Plastibell device led the authors to conclude that this method was less suitable for settings where emergency care is unavailable [14].

Circumcision devices such as the PrePex or Shang Ring have the potential to increase the efficiency of circumcision programs in Africa by faster procedure times allowing greater throughput of clients, lower surgical skill requirements and the potential for task shifting to less highly skilled health personnel such as nurses. The cost-effectiveness of these devices is still unknown [13], [15] since prices for large scale programs have not been finalized. The manufacturer's cost per PrePex device for this study was USD $20.00, but does not include the costs of personnel, other accessories and facilities which are unavailable at time of writing.

Limitations to this study include the fact that this was observational and not a randomized trial, nevertheless findings are consistent with those from other studies. Another potential limitation is that participants who accepted study enrolment could have been self selected with those interested in PrePex being more likely to enroll. This can potentially lead to over estimation of the acceptance of the PrePex device.

In summary, we found that the PrePex device was well accepted in this rural Ugandan population but healing was slower than with conventional surgery, recipients reported an unpleasant odor during the first week, and patient self-removal of the device that can lead to serious complications necessitating emergency surgical intervention was a concern. These results support the WHO TAG [12] recommendations that, while the PrePex device is efficacious and safe in men 18 years and older, clients need to be instructed not to remove the device themselves, clients and providers should be trained to recognize severe complications, and surgical facilities should be available or accessible within 6–12 hours in order to manage these complications. It would be an advantage if the device design could incorporate safe guards against self-removal.
